# Combining Genome and Gene Co-expression Network Analyses for the Identification of Genes Potentially Regulating Salt Tolerance in Rice

**DOI:** 10.3389/fpls.2021.704549

**Published:** 2021-08-26

**Authors:** Panita Chutimanukul, Triono Bagus Saputro, Puriphot Mahaprom, Kitiporn Plaimas, Luca Comai, Teerapong Buaboocha, Meechai Siangliw, Theerayut Toojinda, Supachitra Chadchawan

**Affiliations:** ^1^Center of Excellence in Environment and Plant Physiology, Department of Botany, Faculty of Science, Chulalongkorn University, Bangkok, Thailand; ^2^Program in Biotechnology, Faculty of Science, Chulalongkorn University, Bangkok, Thailand; ^3^Advanced Virtual and Intelligent Computing Research Center, Department of Mathematics and Computer Science, Faculty of Science, Chulalongkorn University, Bangkok, Thailand; ^4^Omics Science and Bioinformatics Center, Faculty of Science, Chulalongkorn University, Bangkok, Thailand; ^5^Genome Center and Department of Plant Biology, University of California Davis Genome Center, UC Davis, Davis, CA, United States; ^6^Molecular Crop Research Unit, Department of Biochemistry, Faculty of Science, Chulalongkorn University, Bangkok, Thailand; ^7^National Center for Genetic Engineering and Biotechnology, National Science and Technology Development Agency, Khlong Luang, Thailand

**Keywords:** transcriptome analysis, gene co-expression network, salt-tolerant genes, rice, clustering co-efficient

## Abstract

Salinity stress tolerance is a complex polygenic trait involving multi-molecular pathways. This study aims to demonstrate an effective transcriptomic approach for identifying genes regulating salt tolerance in rice. The chromosome segment substitution lines (CSSLs) of “Khao Dawk Mali 105 (KDML105)” rice containing various regions of DH212 between markers RM1003 and RM3362 displayed differential salt tolerance at the booting stage. CSSL16 and its nearly isogenic parent, KDML105, were used for transcriptome analysis. Differentially expressed genes in the leaves of seedlings, flag leaves, and second leaves of CSSL16 and KDML105 under normal and salt stress conditions were subjected to analyses based on gene co-expression network (GCN), on two-state co-expression with clustering coefficient (CC), and on weighted gene co-expression network (WGCN). GCN identified 57 genes, while 30 and 59 genes were identified using CC and WGCN, respectively. With the three methods, some of the identified genes overlapped, bringing the maximum number of predicted salt tolerance genes to 92. Among the 92 genes, nine genes, *OsNodulin, OsBTBZ1, OsPSB28, OsERD, OsSub34, peroxidase precursor* genes, and three expressed protein genes, displayed SNPs between CSSL16 and KDML105. The nine genes were differentially expressed in CSSL16 and KDML105 under normal and salt stress conditions. *OsBTBZ1* and *OsERD* were identified by the three methods. These results suggest that the transcriptomic approach described here effectively identified the genes regulating salt tolerance in rice and support the identification of appropriate QTL for salt tolerance improvement.

## Introduction

Salinity is a major environmental stressor that affects rice production worldwide. Salt stress decreases crop yield and limits agricultural productivity (Munns, 2002), particularly in non-irrigated farmlands by triggering two primary effects on plants, osmotic stress, and ion toxicity (Boyer, 1982). In most rice cultivars, the seedling and early booting stages are the most sensitive to salt stress (Lafitte et al., 2007). High concentrations of sodium ions are toxic to most plants (Dionisio-Sese and Tobita, 2000). A combination of ion toxicity and osmotic stress inhibits growth and affects plant development or cause cell death (Hasegawa et al., 2000; Zhu, 2001, 2002). Moreover, these factors affect enzyme activities, which lead to a reduction in photosynthetic rate, metabolism, growth, and development; additionally, pollen germination may also be affected, lowering fertility. These effects contribute to the lower yield of crops exposed to salt stress (Abdullah et al., 2001).

Salt tolerance is a polygenic trait, and although several genes regulating salt tolerance have been identified, there are still some genes regulating salt tolerance in different rice varieties that are yet to be identified. Thai jasmine rice or “Khoa Dawk Mali 105” (“KDML105”) rice is one of the most popular Thai rice cultivars among consumers. The high quality KDML105 grains are produced in rain-fed farms in the northeastern part of Thailand, and the farmlands are characterized by high soil salinity (2–16 dS.m^−1^). Kanjoo et al. (2012) developed a drought tolerant line by generating chromosome substitution lines (CSSLs) in the KDML105 rice genetic background. The introgressions in these CSSLs contain drought-tolerant quantitative trait loci (QTL) on chromosome 1 and were engineered *via* marker-assisted breeding by crossing KDML105 to a drought-tolerant donor, DH212. Chutimanukul et al. (2018a,b). Chutimanukul et al. (2019) reported that CSSL16, a CSSL from this population, exhibited salt tolerance when compared to other CSSLs and KDML105 at the vegetative and seedling stage.

RNA-seq has been widely used to investigate transcriptomes under biotic and abiotic stress conditions in several plants (Song et al., 2014; Garg et al., 2015). High-throughput information can be analyzed to understand plant responses at the transcriptional level using various methods. The gene co-expression network (GCN) is a simplified method used in investigating the biological functions of genes under different conditions using the node degree or hub centrality. GCN analysis was applied to identify the gene modules that regulate drought tolerance (Sircar and Parekh, 2015), salt tolerance (Chutimanukul et al., 2018b), and osmotic stress tolerance (Nounjan et al., 2018). However, this type of network is an undirect graph, which contains nodes corresponding to genes and edges representing neighborhood relations (Stuart et al., 2003; Lee et al., 2004). Recently, the analysis of complex data is being carried out using high-performance computing systems. Consequently, the clustering coefficient method was developed to identify genes in plants or animals exposed to different environment (Zhang and Horvath, 2005).

In the analysis of network topological features, the node degree is one of the most generally used analytical techniques to identify the connection between the number of hub genes and neighboring nodes in the network. The consideration of the important genes can refer to the high number of neighboring nodes. The local density of the connection, referred to as the clustering coefficient (CC), is the measurement of the local density that quantifies the network's tendency of the connections (Watts and Strogatz, 1998; Ravasz et al., 2002). Furthermore, CC was developed from a simple binary network to a weighted network to fulfill the prediction constant degree of any real-world network (Humphries and Gurney, 2008). There have been reports of CC in GCN datasets from yeast and cancer microarrays (Zhang and Horvath, 2005). Moreover, the data analysis of degree on weighted gene co-expression network (WGCN) can be used to construct the signed gene co-expression network to define transcriptional modules (Horvath, 2011). This technique can identify the hub genes in plants or animals subjected to different conditions and the genes responsible for human diseases (Horvath, 2011; Mukund and Subramaniam, 2015; Riquelme Medina and Lubovac-Pilav, 2016).

To perform the expression network analysis for the identification of genes regulating salt tolerance in rice, we used the expression datasets from a single pair of rice lines with similar genetic backgrounds, but different levels of salt tolerance. Therefore, we selected the CSSL population because the lines share a similar genetic background but possess different levels of salt tolerance. To create an expression network, transcriptome datasets of the selected lines at seedling and booting stages were used to identify the major (hub) genes responsible for salt tolerance, as these two stages are the most susceptible to salt stress in rice.

In this study, we compared various CSSLs with different size segments of the putative abiotic stress tolerance genomic region to validate the salt tolerance of CSSL16 at the booting stage. The transcriptome data from leaves at the seedling stage, second leaf, and flag leaf at the booting stage of CSSL16 were analyzed using GCN, CC, and WGCN to predict the major genes responsible for salt tolerance. The expression of some predicted genes was investigated in both salt-tolerant and-susceptible lines.

## Materials and Methods

### Plant Materials

**Figure 1:**
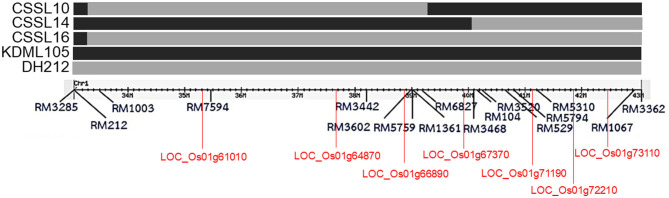
The chromosomal segment substitution line of CSSL10, CSSL14, and CSSL16 with regions between RM1003 and RM3362 markers on chromosome 1. Some genes with putative functions, *Nodulin* (*LOC_Os01g61010*), *BTBZ1* (*LOC_Os01g66890*), *PSB28* (*LOC_Os01g71190*), and *ERD* (*LOC_Os01g72210*), are included.

Rice (*Oryza sativa* L.) seeds of CSSL lines (CSSL10, CSSL14, and CSSL16) with “KDML105” rice genetic background, and their parents (DH212 and KDML105) were obtained from the Rice Gene Discovery Unit (RGDU), National Center for Genetic Engineering and Biotechnology (BIOTEC), Thailand. CSSL16 contained the full segment of the putative salt tolerance region between RM1003–RM3362 (Chutimanukul et al., 2018b), while CSSL10 contained the segment between RM1003–RM6827, and CSSL14 contained the segment between RM3468–RM3362 (Figure 1). The three CSSL lines, CSSL10, CSSL14, and CSSL16, were compared with KDML105 and DH212 for salt stress responses. Then the best CSSL candidate for salt tolerance was selected for transcriptomic analysis.

### Determination of the Photosynthetic Rate and Yield Components of the Lines at Booting Stage

#### Plant Growth Condition

CSSL10, CSSL14, CSSL16, and their parental lines, “KDML105” and DH212 were grown in plastic pots containing soil. We supplied the necessary nutrients by applying Bangsai nutrient solution (1:100) to the soils. At the booting stage, 75 mM NaCl was added to the nutrient solution of the treatment groups, but not to the control group. The addition of NaCl increased the soil EC to 8 dS.m^−1^, thus inducing salt stress. The experiment was performed in randomized complete block design with four replicates. Three plants per replicate were used for collecting the data. Analysis of variance was performed and means were compared with Duncan's multiple range test.

#### Measurement of Physiological Parameters

After 6 days of salt-stress at the booting stage, standard physiological responses, such as net photosynthetic rate (*P*_*n*_), stomatal conductance (*g*_*s*_), internal CO_2_ concentration (*Ci*), transpiration rate (*E*), F_v_/F_m_, and performance index (Pi), were evaluated. In parallel, every 3 days from day 0 to 9 during salt-stress treatment, we classified rice responses using the standard evaluation system (SES) of rice (IRRI, 1996). After 9 days of salt stress, the saline solution was washed out to reduce soil salinity to 2 dS.m^−1^. Plants were then grown until seed harvest and yield components were determined.

At day 6 of salt stress, we measured gas exchange parameters in the middle portion of the flag leaves using a portable photosynthesis system (LI-6400 XT; LI-COR, Lincoln, NE). We used three plants per group as a replicate. The leaves were examined under the following conditions: 500 mmol m^−2^ s^−1^ air flow per unit leaf area, 1,200 mol m^−2^ s^−1^ photosynthetically active radiation (PAR) at leaf surface, leaf temperature ranged from 31.0 to 35.0°C, and a CO_2_ concentration of 380 mol mol^−1^.

F_v_/F_m_ and Pi were measured according to the recommended procedures of FMS 2 (Hansatech, King's Lynn, UK). Leaves were dark-adapted for 40 min using dark-adapted leaf clips before measurement.

#### Experimental Design and Statistical Analysis

The study was laid out in a completely randomized design (CRD), with four replicates per treatment group (samples from three plants in a group constituted a replicate). Data of physiological parameters were subjected to analysis of variance (ANOVA) and significant means were compared using Duncan's multiple range tests (DMRT) by using SPSS version 21 (IBM Corp, Armonk, USA). Values were considered statistically significant at *p* < 0.05.

### Identification of the Putative Salt Tolerant Genes *via* Transcriptome Analysis

#### RNA Extraction and Sequencing

To identify the genes regulating salt tolerance in rice, we focused on transcriptome analysis of CSSL16, which had the highest salt tolerance in the seedling and booting stages. Three replicates were used for each condition (CSSL16 grown under normal condition and under salt stress (75 mM NaCl treatment), respectively). Leaf tissues were collected at the seedling and booting stages. We harvested leaf samples from 21 days old seedlings after 0 and 2 days of salt stress, while flag leaves and second leaves were harvested at the booting stage on days 0 and 3. Leaves from the seedlings, flag leaves, and second leaves of untreated plants were used as the control. Three biological replications were conducted for this experiment. Total RNA was extracted from the leave samples using plant RNA purification reagent (Invitrogen, USA), and contaminated genomic DNA was removed with DNaseI (Invitrogen). cDNA libraries were constructed using the KAPA Stranded RNA-Seq Library Preparation Kit from Illumina® (Kapa Biosystem, USA). All short reads with a size of ~300 bp were selected and connected with adaptors. Thereafter, all fragments were enriched by PCR for 12 cycles. The cDNA libraries were sequenced using Illumina Next-Generation sequencing (Illumina, USA).

For transcriptome analysis, all short-sequence reads were classified into the right category and QC was performed using a pipeline created by Missirian et al. (2011). The transcriptome sequences were uploaded to the NCBI database with BioProject ID, PRJNA507040. The sequence reads were aligned and mapped to the rice genome database (Ouyang et al., 2007) using Bowtie 2 (Langmead and Salzberg, 2012). The DESeq program (version 1.24.0) was used to identify differentially expressed genes (Anders and Huber, 2010). Genes with *p*-value <0.01 were identified as differentially expressed genes.

### Identification of Marker Genes by GCN and CC Analysis

The read count of the RNA-Seq was analyzed and normalized using the DESeq package in software R (Anders and Huber, 2010). We constructed the gene co-expression network of the rice lines under normal and salt stress conditions at the growth stages following the method of Suratanee et al. (2018), and these constructs were combined as whole-state networks. The expression levels of whole-state networks were mixed. The edges in the network were recognized by calculating and selecting gene pairs with highly correlated (*r* ≥ 0.9) levels of expression. Node degree is the number of edges connected to a node in a network, and clustering coefficient is a measure of the proportion of true connections and the number of all possible connections among neighbors of a gene node. The nodes represent the investigated genes, and the edges represent the significant co-expression level of any of the gene pairs. GCN identifies genes by using the degree or hub centrality. The clustering coefficient (CC) is a common measure of the true proportion of the link between the gene nodes and neighbors. The original clustering coefficient (small-world network) by Watts and Strogatz (1998) is as follows:

(1) $$\begin{array}{l} C\left(i\right)=\frac{\sum_{j}\sum_{q\neq j}\left(a_{\left(ij\right)}a_{\left(iq\right)}a_{\left(jq\right)}\right)}{k_{i}\left(k_{i}-\;1\right)} \end{array}$$

*C(i)* varies from 0 to 1. *a*_*ij*_ is a binary value from the connection between node *i* and node *j*. The degree of node *i* is *k*_i_. If all neighbors of *i* are themselves connected to another, CC equals 1, and if the neighbors of *i* do not connect to each other, CC equals 0. Based on a real-world network, their nodes are mostly connected with some level of strength connections or weights. Moreover, the clustering coefficient for a weighted graph was constructed from the total weights of the neighbors (Onnela et al., 2005).

### Identification of Marker Genes by Weighted Co-expression Network (WGCN)

For WGCN, the connection of the network has its own values as a binary network of 0 or 1. Therefore, a weighted degree is the sum of all edges connecting the given node and neighbors. According to Onnela et al. (2005), a weighted graph of the clustering coefficient is obtained by taking the geometric mean of the total weights of its neighbors. Moreover, these connection weights can be positive or negative. While 0 represents no connection with neighbors, 1 represents the highest connection with all neighbors. The formula for using the real weights in the network is as follows:

(2) $$\begin{array}{l} C_{\text{realweight}}\left(i\right)=\frac{\sum_{j}\sum_{q\neq j}|w_{\left(ij\right)}w_{\left(iq\right)}w_{\left(jq\right)}{|}^{\frac{1}{3}}}{k_{i}\left(k_{i}-\;1\right)} \end{array}$$

The weight of the edge connecting nodes *i* and *j* is w_*ij*_. The connection weights can be categorized as positive or negative. The value of *C*_*realweight*_*(i)* is distributed in the range [0, 1], where 0 means that there were no neighbors to connect to each other, and 1 means that there were high connections with neighbors. This formula was used to calculate the clustering coefficient for the real weights in the network, while the original formula was performed using a cut-off for the weight estimation into a binary class.

**Figure 2:**
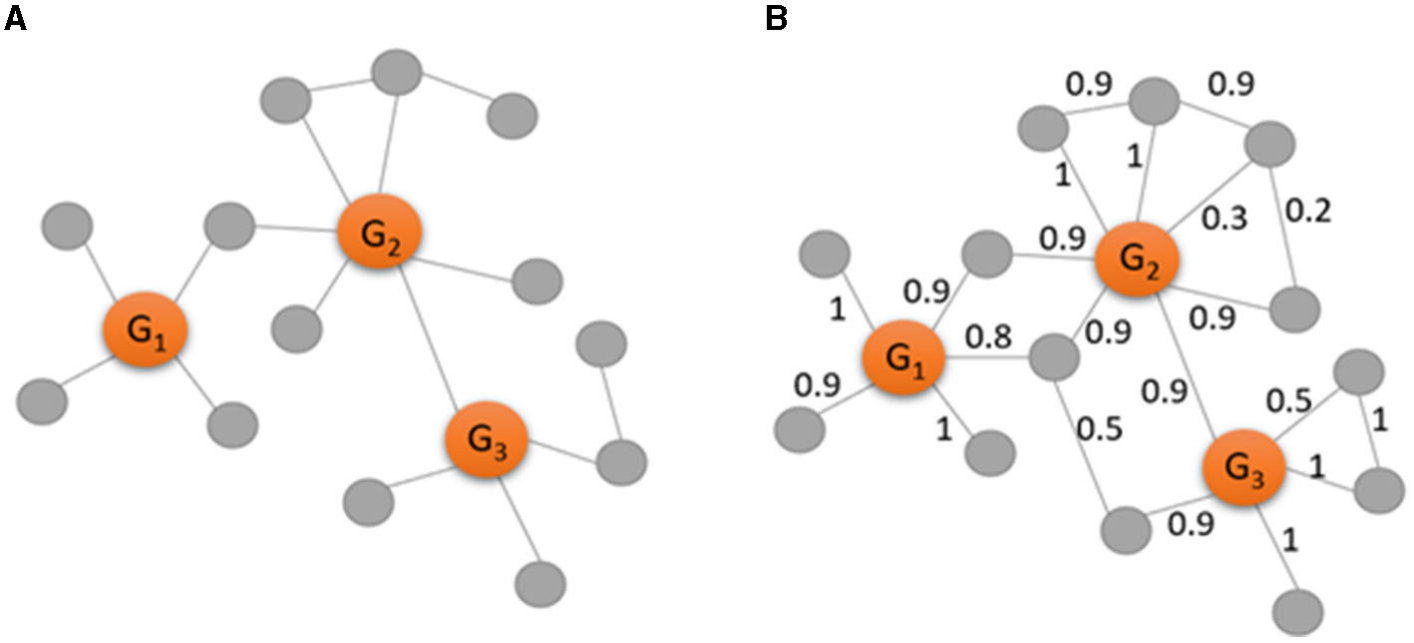
Examples of a binary gene co-expression network **(A)** and a weighted gene co-expression network **(B)** consisting of three observed genes (in orange) and 13 genes (in gray). The edges in the network **(A)** are recognized by calculating and selecting gene pairs with highly correlated (*r* ≥ 0.9), while the edges in the network **(B)** are weighted by the absolute values of the correlation.

To clarify the analysis of GCN, CC, and WGCN, Figure 2 shows an example of a gene co-expression network in the form of a binary network (Figure 2A) and in the form of a weighted network (Figure 2B). Gene identification by GCN analysis involves calculating the degree for each gene in the binary network in Figure 2A. Then, the highly connected nodes are recognized as marker genes. Therefore, G_1_ with degree of 4, G_2_ with degree of 6, and G_3_ with degree of 4 have more connections than the other genes and are identified as important markers. On the other hand, gene identification of CC explores the possibility of connections among the neighbors of a certain node. There are no connections among the neighbors of G_1_ and among the neighbors of G_3_, while there is one connection among the neighbors of G_2_. Therefore, the CC values of G_1_ and G_3_ are zero while the CC value of G_2_ is 1/15 since 15 is the total number of all possible connections among the six neighbors.

Gene identification by WGCN involves the direct calculation of a weighted degree, that is the sum of all edge weights for a certain node in Figure 2B. With the use of weighted network, there are more edges with known strength as more information needs to be considered. Thus, the weighted degree of G_1_ is 4.6, the weighted degree of G_2_ is 5, and the weighted degree of G_3_ is 4.3. Comparing with the degree values above, the weighted network indicates that G_1_ is more important than G_3_ while they have the same level of importance in the binary network.

### Validation of the Salt Tolerant Candidate Genes by Gene Expression Analysis

To validate regulation of salt-tolerance candidate genes by qRT-PCR, CSSL16, which had the highest salt tolerance at the seedling and booting stages, was compared with KDML105. The seeds of CSSL16 and KDML105 were soaked in water to induce germination. After 7 days, the seedlings were transplanted to nutrient solution (Udomchalothorn et al., 2014) with three replicates (three seedlings per replicate). Subsequently, after 7 days, the seedlings were transferred to nutrient solution without NaCl (control) and nutrient solution containing 75 mM NaCl (treatment group). Seedlings were harvested after salt stress treatment for 0, 3, 6, 12, 24, and 48 h for the early response and for the late response, seedlings were harvested on days 0, 3, and 6 of treatment.

### Gene Expression Analysis

Total RNA was extracted from the shoots of seedlings from the control and treatment groups using GENEzol GZR100 (Geneaid Biotech, Taiwan). The RNA was treated with DNase I (Thermo Scientific, USA) and converted into cDNA. cDNA synthesis was performed using an Accupower RT premix (Bioneer Inc., Alameda, USA). The synthesized cDNA was used as template for the PCR. qRT-PCR was conducted using Luna Universal qPCR master mix M3003L (New England Biolabs Inc., USA).

Quantitative RT-PCR reactions were conducted on three technical replicates for each sample. No template (NTC) was used as a negative control, and *EF-1*α primers (Chutimanukul et al., 2018b) were used as an internal control to standardize the equal template in the reaction. Gene sequences were obtained from the rice genome database (Ouyang et al., 2007) and then submitted to Primer3 to generate specific primers for the nine selected genes (Table 1). Relative gene expression was determined by qRT-PCR. The PCR conditions were as follows: an initial denaturation step at 95°C for 60 s, followed by 35 cycles of denaturation at 95°C for 15 s, annealing steps with the temperature shown in Table 1 for 30 s, and continued with an extension step at 75°C for 30 s. The melt curve and plate read were set at 60–94°C with increasing temperature at the rate of 5°C per 5 s. Average cycle threshold (Cq) values of all genes were normalized to the level of *EF-1*α reference genes in the same sample and then used to measure relative gene expression by following the ΔΔCt method as described by Pfaffl (2001). The gene expression analysis was interpreted based on the relative expression levels, and SPSS software was used for the analysis of variance (*p* < 0.05).

**Table 1:** Quantitative RT-PCR rice primers.

**Name/annotation**	**Sequence 5'à3'**		**Product size**	**Position**	**Annealing temperature**
*LOC_Os01g61010 (Nodulin)*	FW	CCGCGAAAAGTGGCTACTCCA	101 bp	1,179–1,282	60.0°C
	RV	AAAGAAGTCCCGCTGGTTGAG			
*LOC_Os01g64870*	FW	CGAGCAGTTTGCCAGGTTGAAT	183 bp	974–1,156	61.5°C
	RV	AGCCTTTGGAATGCAAGCTCCT			
*LOC_Os01g66890*(*BTBZ1*)	FW	TTCCTGCCTGCAAGGGCATC	172 bp	1,108–1,280	61.5°C
	RV	TCCTTGAAATGCCTACAGAGGGG			
*LOC_Os01g67370*	FW	GGCGGATTTACCGAACATATTTGA	173 bp	260–432	60.5°C
	RV	TGTCAGCCAGGAAGGTTGGA			
*LOC_Os01g72210* (*ERD*)	FW	GGTTCTAACAAGCTTTGGGTGC	141 bp	562–703	61.5°C
	RV	TTGGTCAGGCCGTTTCCTGT			
*LOC_Os01g71190 (PSB28)*	FW	GATGCCCCGCAGGTTCGTC	170 bp	218–387	60.0°C
	RV	GGTGCCCTGGATGAACTGGA			
*LOC_Os01g73110*	*FW*	CCGATGGTGATGGTTGGCTG	180 bp	160–339	61.0°C
	RV	CCGATCCAGCTTGCGCTCT			
*LOC_Os04g03050* (*Sub34*)	FW	TGTGGTTATCACCTTGGGCG	124 bp	1,164–1,287	61.0°C
	RV	ATTGTCGGCATTGCAGTCGT			
*LOC_Os06g46799* (*Peroxidase*)	FW	CCTCTCCTCCTTCCAGAGCAA	97 bp	629–725	61.0°C
	RV	GCTGAACGAGTTGCAGTGCG			
*EF1α*	FW	ATGGTTGTGGAGACCTTC	127 bp	1,326–1,435	60.0°C
	RV	TCACCTTGGCACCGGTTG			

### Analysis of Arabidopsis Mutant Lines for Salt Stress Responses

The selected mutant seeds were ordered from Arabidopsis Biological Resource Center (ABRC). The homozygous mutant lines were screened according to SALK T-DNA primer design. The homozygous mutant lines used in this experiment were *bt3, psb28, AT5G45310, sbt3.3, sbt3.4*, and *per3* mutants. Col-0 wild type (WT) was used as a control. The evaluation of salt stress response was performed with complete randomized design with three replicates. Each replicate contained 20 seedlings. Mutant lines and WT seeds were sterilized and germinated for 7 days after stratification at 4°C for 48 h. Then, 7 day-old seedlings were transferred to the freshly prepared MS medium with or without 100 mM NaCl addition. After 7-day incubation under light intensity of 35 mmol.m^−2^.s^−1^, 16/8 light/dark cycle at 22 °C, dry weight was measured with 15 plants per treatment. Photosynthetic pigment contents were determined from 5 plants per treatment according to Wellburn (1994). The absorbance at A_470_, A_646.8_, and A_663.2_ were measured to determine Chlorophyll *a*, chlorophyll *b* and carotenoid contents by using the following equations:

(3) $$\begin{array}{l} Chlorophyll\;a\;\left(Chl\;a\right)\;content=12.25A_{663.2}-2.79A_{646.8} \end{array}$$

(4) $$\begin{array}{l} Chlorophyll\;b\;\left(Chl\;b\right)\;content=21.5A_{646.8}-5.1A_{663.2} \end{array}$$

(5) $$\begin{array}{l} Totalcarotenoids=\left(100A_{470}-1.82Chla-85.02chlb\right)/198 \end{array}$$

### Putative Promoter Analysis

The putative promoter region (2 kb upstream from coding region) of *OsBTBZ1* gene of KDML105 and CSSL16 was retrieved from PRJNA659381. Sequence alignment was performed by using Needle tool *via* EMBOSS. Cis-elements were searched against PLACE database (Higo et al., 1999).

## Results

### CSSL16 Sustained Photosynthetic Responses Under Salt Stress at Booting Stage

**Figure 3:**
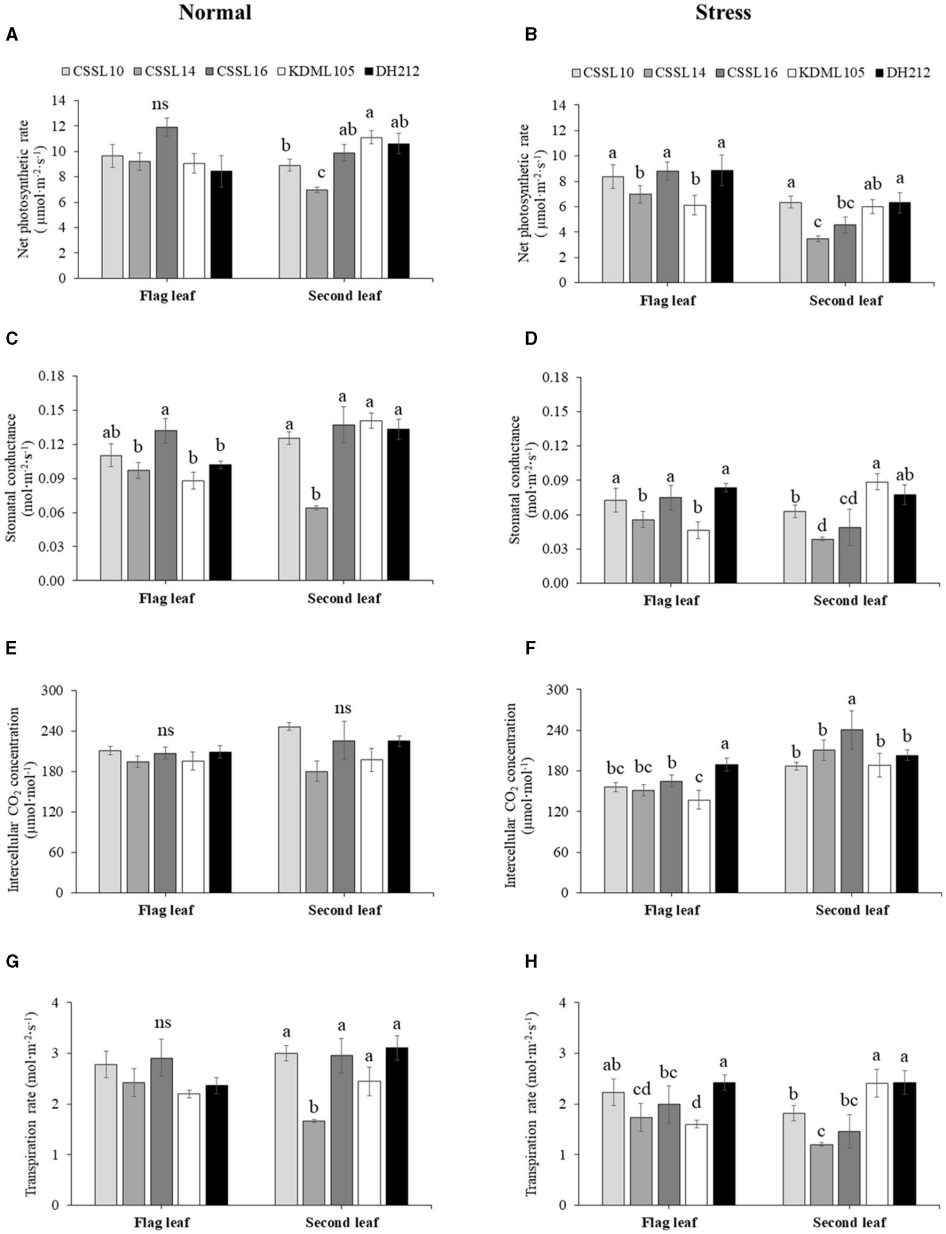
Gas exchange parameters, net photosynthesis rate [Pn, **(A,B)**], stomatal conductance [*g*_*s*_, **(C,D)**], internal CO_2_ concentration [*Ci*, **(E,F)**], and transpiration rate **(E,G,H)** of flag leaves and second leaves of CSSL10, CSSL14, CSSL16, “KDML105,” and DH212 under normal and salt stress conditions. Values are represented as mean ± SE (*n* = 4). Different letters above bars indicate significant difference between lines at *p* < 0.05. “ns” indicates no significant difference.

The physiological study showed that the net photosynthesis rate (*Pn*) of the flag leaves of the rice lines under normal grown condition was not significantly different (Figure 3A). However, the *Pn* of the second leaves of the lines were significantly different, with the second leaves of “KDML105” recording the highest *Pn* values, while the second leaves of CSSL14 grown under normal conditions had the lowest *Pn* values.

Salt stress caused a decrease in the *Pn* of the flag leaf and second leaf of the lines (Figure 3B). The flag leaves of CSSL10, CSSL16, and DH212 had significantly higher *Pn* than those of “KDML105” and CSSL14, while the second leaves of CSSL10 had similar *Pn* values to those of “KDML105” and DH212. A similar response was also found in stomatal conductance (Figures 3C,D). The *Ci* levels of rice grown under normal conditions were not significantly different; contrarily, the *Ci* levels of both flag leaves and second leaves of rice lines grown under salt stress were significantly different with the second leaves of “CSSL16” recording the highest *Ci* level (Figures 3E,F). The transpiration rate of these plants was consistent with their *g*_*s*_ (Figures 3G,H).

**Figure 4:**
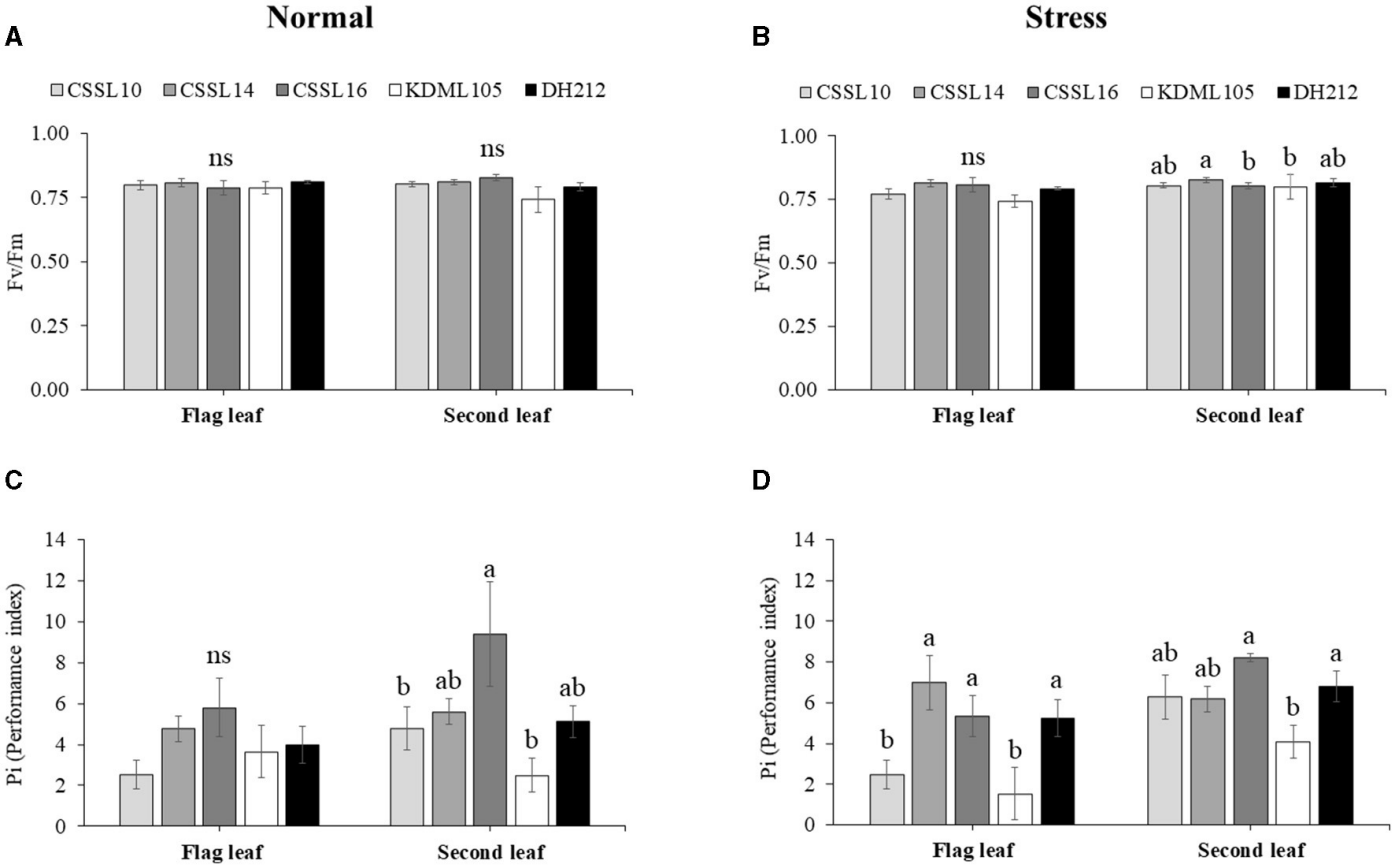
Maximum PSII efficiency (F_v_/F_m_) **(A,B)** and Performance index (Pi) **(C,D)** of flag leaves and second leaves in CSSL10, CSSL14, CSSL16, “KDML105.” and DH212 under normal and salt stress conditions. Values are represented as mean ± SE (*n* = 4). Different letters above bars indicate significant difference between lines at *p* < 0.05. “ns” indicates no significant difference.

**Figure 5:**
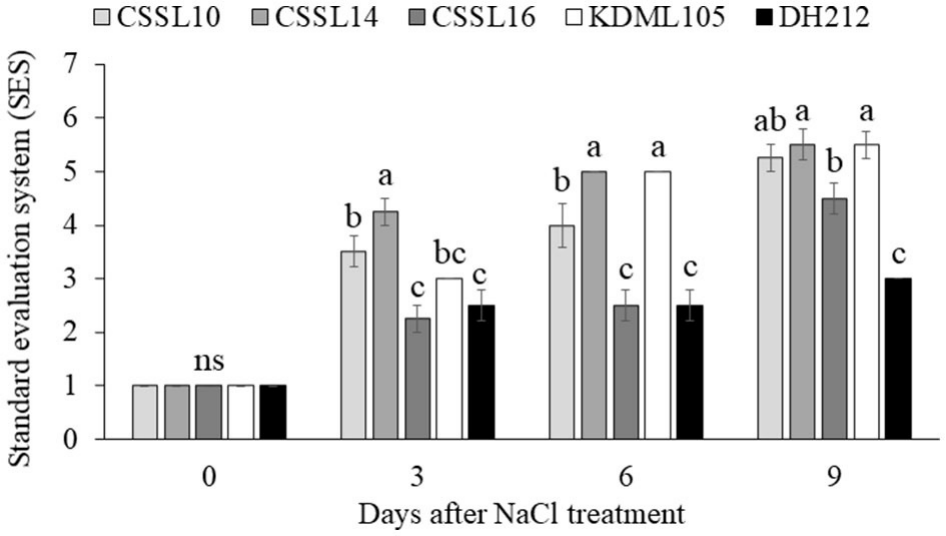
Standard evaluation system (SES) determined from the appearance of plants under salt stress condition for 0, 3, 6, and 9 days. Values are presented as mean ± SE (*n* = 4). Different letters above bars indicate significant difference between lines at *p* < 0.05. “ns” indicates no significant difference.

Salt stress did not affect the PSII efficiency (F_v_/F_m_) of the flag leaves (Figures 4A,B). Additionally, the Pi's of the flag leaves were not significantly different under normal growth condition; contrarily, salt stress significantly affected the Pi's of the flag leaves, with CSSL14 recording the highest Pi, while “KDML105” recorded the lowest. The second leaves of CSSL16 recorded the highest Pi both under normal growth condition and under salt stress, while the second leaves of “KDML105” had the lowest Pi both under normal growth condition and under salt stress. Overall, the Pi's of the second leaves of the rice lines were significantly different both under normal growth conditions and under salt stress (Figures 4C,D). During the first 6 days and after 9 days under salt stress conditions (Figure 5), CSSL16 and DH212 had significantly lower SES than the other lines.

### CSSL16 Had Higher Yield Components Than That Did “KDML105” and Other CSSLs

After exposing the rice seedlings to salt stress at 8 dS.m^−1^ for 9 days, soil salinity was reduced to 2 dS.m^−1^ and the plants were grown under this condition until grain harvest. The yield components of the different lines were determined after harvest (Table 2). Results showed that rice lines with KDML105 genetic background recorded higher tiller numbers per plant than the corresponding, introgression-free line DH212. Salt stress decreased tiller numbers per plant, panicle numbers per plant, panicle length, total seed number, and number of filled grains per plant. Moreover, shoot fresh weight, dry weight, and height were affected by salt stress (Table 2). CSSL16 had the highest tiller numbers per plant, panicle number per plant, total seed number per panicle, filled grain, and seed number per plant, compared to the other lines. Based on gas exchange parameters, PSII efficiency and yield component, CSSL16 was the most tolerant line under high salt stress at the booting stage. This suggested that the presence of the whole QTL region was required to achieve the best tolerance, implicating the action of two or more genes Therefore, CSSL16 was chosen for transcriptome analysis.

**Table 2:** Yield components of CSSL10, CSSL14, CSSL16, “KDML105,” and DH212 grown under normal or salt stress conditions (8 dS.m^−1^) at booting stage for 9 days.

**Yield components^†^**	**Condition**	**Rice lines**					***F*-test**
		**CSSL10**	**CSSL14**	**CSSL16**	**KDML105**	**DH212**	
Tiller number per plant	Normal	14.25 ± 0.85^a^	15 ± 1.77^a^	16.75 ± 0.63^a^	13.25 ± 0.85^a^	8.5 ± 1.19^b^	^*^
	Salt stress	10.25 ± 0.85^bc^	12 ± 0.71^ab^	14 ± 1.08^a^	12.25 ± 0.47^ab^	7.75 ± 1.03^c^	^*^
Panicle number per plant	Normal	9.25 ± 0.85^b^	9.75 ± 0.85^b^	14.5 ± 0.29^a^	10.25 ± 0.95^b^	8.5 ± 1.55^b^	^*^
	Salt stress	7.75 ± 1.11	9.25 ± 0.95	11 ± 1.47	9.75 ± 1.11	7.5 ± 1.19	ns
Panicle length (cm)	Normal	27.78 ± 0.54^a^	26.14 ± 0.33^b^	24.98 ± 0.29^c^	25.96 ± 0.17^bc^	27.31 ± 0.21^a^	^*^
	Salt stress	24.31 ± 0.17^b^	24.12 ± 0.36^b^	23.49 ± 0.26^b^	21.46 ± 0.53^c^	25.92 ± 0.26^a^	^*^
Total seed per panicle	Normal	130.25 ± 3.94^bc^	116.75 ± 4.31^c^	152.25 ± 3.68^a^	124.25 ± 5.78^c^	143.75 ± 4.40^ab^	^*^
	Salt stress	121.50 ± 4.13^bc^	114.75 ± 1.75^c^	142.75 ± 4.05^a^	113.5 ± 3.10^c^	134.75 ± 2.63^ab^	^*^
Filled grains per plant	Normal	91.75 ± 0.48^c^	94.5 ± 2.10^c^	130.25 ± 2.25^a^	109.75 ± 5.20^b^	114.25 ± 4.85^b^	^*^
	Salt stress	74.25 ± 2.50^d^	75 ± 1.68^d^	117 ± 3.03^a^	90.25 ± 2.29^c^	98.25 ± 2.87^b^	^*^
100 Seeds weight (g)	Normal	1.89 ± 0.07^b^	1.90 ± 0.10^b^	1.92 ± 0.04^b^	2.20 ± 0.10^a^	2.08 ± 0.02^ab^	^*^
	Salt stress	1.14 ± 0.15^b^	1.67 ± 0.08^a^	1.65 ± 0.07^a^	1.85 ± 0.07^a^	1.55 ± 0.16^a^	^*^
Plant height (cm)	Normal	178.25 ± 2.62^a^	171.75 ± 5.21^ab^	156 ± 8.95^b^	121.25 ± 3.35^c^	164.75 ± 4.19^ab^	^*^
	Salt stress	152.5 ± 2.75^a^	160 ± 5.05^a^	139 ± 2.68^b^	109.5 ± 0.65^c^	152.75 ± 2.56^a^	^*^
Shoot fresh weight (g)	Normal	179.25 ± 8.01^a^	147.5 ± 10.13^ab^	157.7 ± 10.40^ab^	116.22 ± 11.54^b^	132.19 ± 22.07^b^	^*^
	Salt stress	138.5 ± 7.053	118.25 ± 11.44	125.75 ± 12.30	86.5 ± 13.37	117.25 ± 16.12	ns
Shoot dry weight (g)	Normal	28.74 ± 2.19	28.67 ± 2.10	32.76 ± 0.95	24.56 ± 1.27	29.07 ± 2.27	ns
	Salt stress	24.49 ± 1.41^bc^	27.39 ± 1.02^ab^	29.01 ± 0.53^a^	22.13 ± 0.88^c^	27.94 ± 1.71^ab^	^*^

† *Values are represented as mean ± SE (n = 4). Different letters indicate significant difference between lines at p < 0.05. “ns” indicates no significant difference*.

* *Significant difference at p < 0.05*.

### Transcriptomics Profile of CSSL16 Rice at Seedling and Booting Stages

To identify genes regulating salt tolerance in rice, we analyzed the transcriptome of three seedling leaves, and from the flag and second leaves of CSSL16 plants exposed to normal growth condition and salt stress, respectively. Gene expression was examined by RNA sequencing of the leaves of seedlings at 0 and 2 days of treatment. At the booting stage, RNA sequencing was performed from flag leaf and second leaf samples at 0 and 3 days of treatments. We identified 511 differentially expressed genes in the leaves of the seedling, while 520 and 584 differentially expressed genes were identified in the second leaf and flag leaf, respectively (Supplementary Files 1, 2). More than 50% of the differentially expressed genes were downregulated by salt stress at the seedling stage and in the flag leaves at the booting stage. Contrarily, <50% of the differentially expressed genes were downregulated by salt stress in the second leaf.

We used the ClueGo tool to screen for gene ontology (GO) terms that were significantly enriched by the DEGs. The results showed that genes enriched in biological processes, such as response to inorganic substances, oxygen-containing compounds, alcohol, heat, and temperature stimulus were downregulated in the leaves of the seedlings, while the genes involved in cell wall biogenesis, cellular glucan metabolism, and glucan metabolism were upregulated (Supplementary Figure 1). We compared the transcriptomes of the second leaf before and after 3 days of salt stress. The GO enrichment analysis of the second leaf indicated a significant upregulation of genes regulating temperature and heat responses, and the sizes of cellular components and anatomical structures (Supplementary Figure 2), while genes enriched in cellular chemical homeostasis and chemical homeostasis were downregulated. When the plants were exposed to salt stress, the upregulated genes were enriched in response to heat and temperature stimulus (Supplementary Figure 3).

### Combining the Gene Co-expression Network Analysis With SNP Information Can Identify Salt Tolerant Genes

The co-expression networks under salinity and normal conditions were constructed by calculating the correlation of the expression levels of DEGs in the plants (leaves of the seedlings, flag leaves, and second leaves). Genes that were highly correlated (*r* > 0.9) under normal condition were used to construct the normal-state network. Similarly, genes that were highly correlated under salinity stress were used to construct the salinity-state network. We found 579 DEGs in the normal-state network and 573 DEGs in the salinity-state network. The results showed that the network created from expression data under normal conditions had higher number of nodes, edges, connection per node, and average degree than those of the network created from the expression data under the salt stress condition. The genes involved in salt tolerance were selected from genes with high connections per node under salt stress conditions and low connections per node under normal conditions. Fifty-seven candidate genes (Supplementary File 2) were selected. Most of the selected genes were on chromosome 1. Four of them, *LOC_Os01g64870, LOC_Os01g66890, LOC_Os01g67370*, and *LOC_Os01g72210* were located in the salt/drought tolerant QTL reported by Kanjoo et al. (2012). *LOC_Os01g72210* and *LOC_Os01g67370* encoded unknown expressed proteins, while *LOC_Os01g66890* was annotated as *BTBZ1* and *LOC_Os01g72210*, was annotated as a protein part of the early response to dehydration (*ERD*) protein. Both *BTBZ1* and *ERD* displayed SNPs between CSSL16 and “KDML105” in the promoter, 5'UTR, exons, introns, and 3'UTR.

We analyzed the distributions of the clustering coefficients for the binary network by comparing a dense local cluster between salt stress and normal conditions. The clustering coefficient analysis identified 30 genes involved in salt tolerance (Supplementary File 3). Four genes were located in the salt/drought tolerant QTL (Kanjoo et al., 2012), *LOC_Os01g61010, LOC_Os01g66890* (*BTBZ1*)*, LOC_Os01g72210* (*ERD*), and *LOC_Os01g73110*. The CC analysis identified *BTBZ1* and *ERD*, which were also identified by GCN analysis. *LOC_Os01g61010* was annotated as encoding a *Nodulin*, while *LOC_Os01g73110* encoded an unknown expressed protein.

Furthermore, we identified 59 genes using weighted co-expression network analysis (Supplementary File 2). *LOC_Os01g64870, LOC_Os01g66890* (*BTBZ1*)*, LOC_Os01g71190, LOC_Os01g72210* (*ERD*), and *LOC_Os04g03050* were located in the salt/drought QTL (Koyama et al., 2001; Kanjoo et al., 2011, 2012). Moreover, three out of the five genes (*LOC_Os01g64870, BTBZ1*, and *ERD*) were identified by both the co-expression network and clustering coefficient analyses. The other three genes included *LOC_Os01g71190* (*PSB28*), which was annotated to encode the protein involved in photosystem II reaction center, while *LOC_Os04g03050* and *LOC_Os06g46799* encoded subtilisin (*OsSub34*) and peroxidase precursor, respectively.

**Figure 6:**
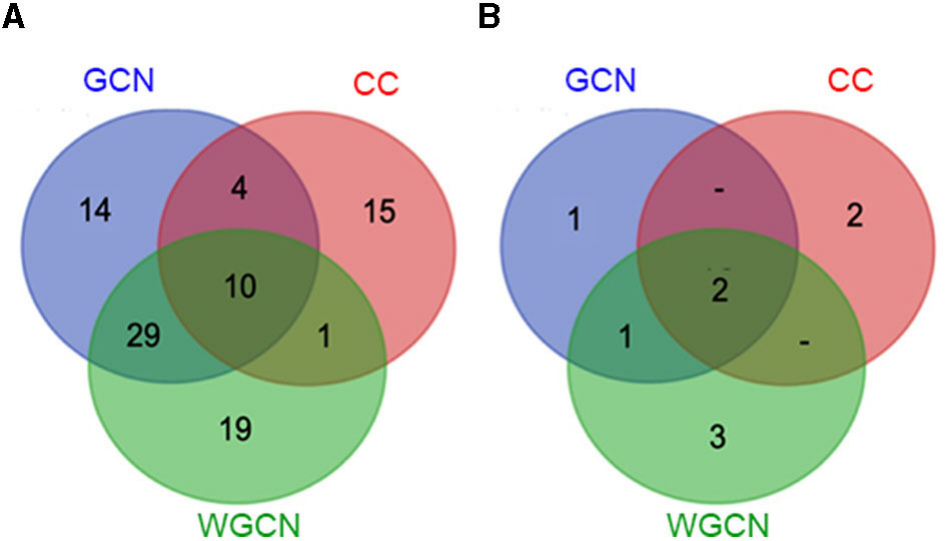
Venn diagram **(A)** showing the number of salt-responsive genes from co-expression network analysis (blue circle), Clustering coefficient analysis (red circle), weighted co-expression network analysis (green circle), and Venn diagram **(B)** showing number of salt-responsive genes containing the SNPs in each method analysis.

**Figure 7:**
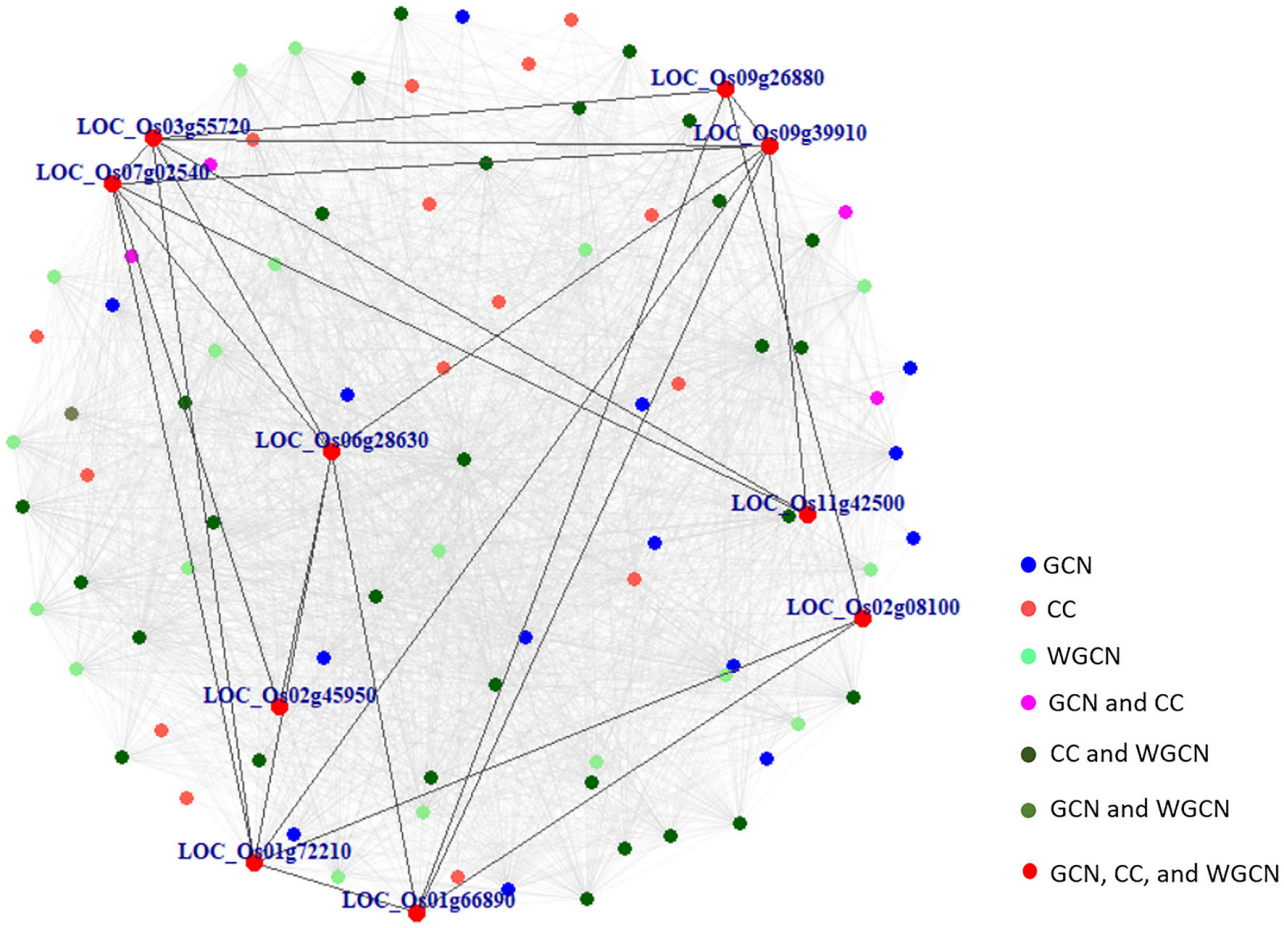
The co-expression network of 92 marker genes identified by GCN, CC, and WGCN. Ten genes in red were detected by all methods and the connections among them were shown in darked lines. All gray lines represent the connections among these 92 marker genes.

Figure 6A displays a Venn diagram of the genes identified using the three network analyses. The blue, red, and green circles included genes identified by GCN, CC, and WGCN, respectively (Figure 6A). In total, we identified 92 genes using the three methods. Among the genes, 10 were identified by each of the three methods (Table 3). The co-expression network of 92 genes identified by GCN, CC, and WGCN is shown in Figure 7. The 10 genes, identified by these three techniques (GCN, CC, and WGCN), are displayed as red circles.

**Table 3:** Salt-tolerant genes consistently predicted by GCN, CC, and WGCN.

**Locus**	**Annotation**
LOC_Os01g66890 (*BTBZ1*)	BTBZ1—Bric-a-Brac, Tramtrack, and Broad Complex BTB domain with TAZ zinc finger and Calmodulin-binding domains, expressed
LOC_Os01g72210 (*ERD*)	Early-Responsive to Dehydration protein-related, putative, expressed
LOC_Os02g08100	AMP-binding domain containing protein, expressed
LOC_Os02g45950	cytochrome b_6_f complex subunit, putative, expressed expressed protein
LOC_Os03g55720	Cytochrome b_6_f complex subunit, putative, expressed
LOC_Os06g28630	Expressed protein
LOC_Os07g02540	HLS, putative, expressed
LOC_Os09g26880	Aldehyde dehydrogenase, putative, expressed
LOC_Os09g39910	ABC transporter, ATP-binding protein, putative, expressed
LOC_Os11g42500	Dirigent, putative, expressed

Using SNPs found in CSSL16 and “KDML105,” the number of genes identified by GCN, CC, and WGCN were 4, 4, and 6, respectively (Figure 6B). Together with the three methods of transcriptome analysis and SNP information of the salt tolerant and susceptible lines, we identified nine genes, which were responsible for salt tolerance in rice (Figure 6B and Table 4). Two out of these genes, which are *LOC_Os01g66890* (*BTBZ1*) and *LOC_Os01g72210* (*ERD*), contain SNPs between CSSL16 and “KDML105” rice. In addition, these two genes are connected to each other in the network (Figure 7). We hypothesize that the nine genes were responsible for the salt tolerance of CSSL16 compared with KDML105 rice.

**Table 4:** Putative salt tolerance genes predicted by GCN, CC, and WGCN containing SNPs between CSSL16 and “KDML105” rice.

**Locus**	**Annotation**	**Types of network analysis**	**Orthologous gene in Arabidopsis**
*LOC_Os01g61010* (*Nodulin*)	Nodulin, putative, expressed	CC	-
*LOC_Os01g64870*	expressed protein	GCN, WGCN	*AT1G71240*
*LOC_Os01g66890* (*BTBZ1*)	BTBZ1—Bric-a-Brac, Tramtrack, and Broad Complex BTB domain with TAZ zinc finger and Calmodulin-binding domains, expressed	GCN, CC, WGCN	*AT1G05690* (*BT3*)
*LOC_Os01g67370*	Expressed protein	GCN	*AT3G59300*
*LOC_Os01g71190*	Photosystem II reaction center PSB28 protein, chloroplast precursor, putative, expressed	WGCN	*AT4G28660* (*PSB28*)
*LOC_Os01g72210* (*ERD*)	Early-responsive to dehydration protein-related, putative, expressed	GCN, CC, WGCN	*AT3G54510* (*ERD4*)
*LOC_Os01g73110*	Expressed protein	CC	*AT5G45310*
*LOC_Os04g03050*	OsSub34—Putative Subtilisin homolog, expressed	WGCN	*AT1G32940* (*SBT3.5*) *AT1G32950* (*SBT3.4*) *AT1G32960* (*SBT3.3*) *AT4G10510* *AT4G10540* (*SBT3.8*) *AT4G10550*
*LOC_Os06g46799*	Peroxidase precursor, putative, expressed	WGCN	*AT1G05260* (*PER3*)

### Significantly Different Expression Levels of the Candidate Genes in CSSL16 After Salt-Stress Treatment

**Figure 8:**
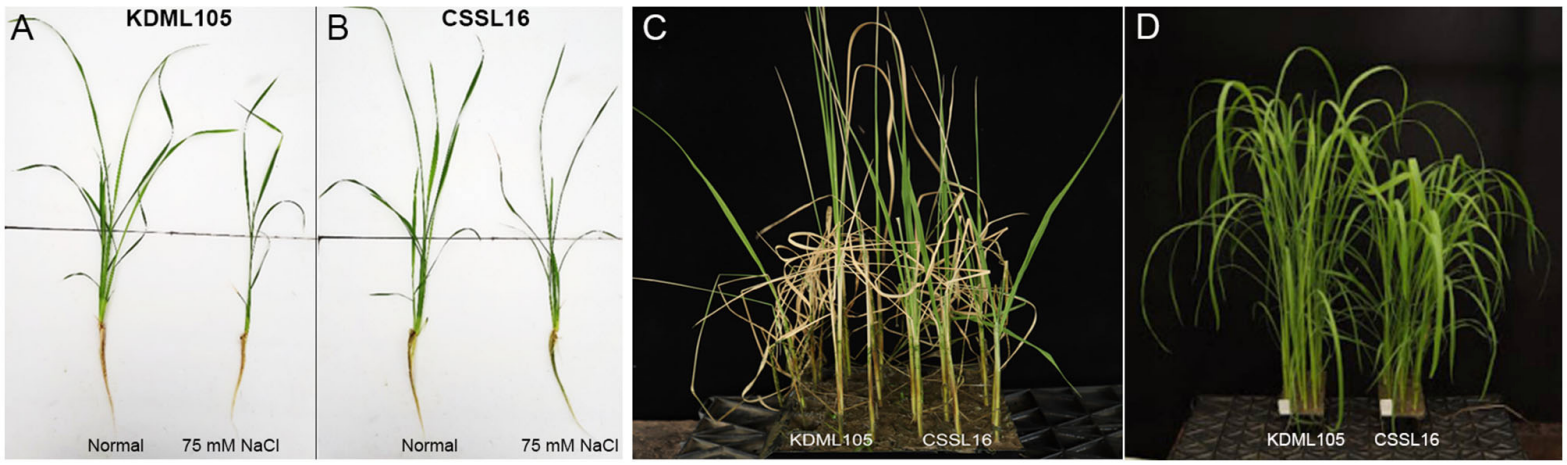
Fourteen day-old KDML105 and CSSL16 seedlings after growing in nutrient solution in nomral condition or supplementaed with 75 mM NaCl for 6 days **(A,B)** and the seedlings that were soil-grown and treated with 75 mM for 12 days **(C)** or grown in normal condition **(D)**.

**Figure 9:**
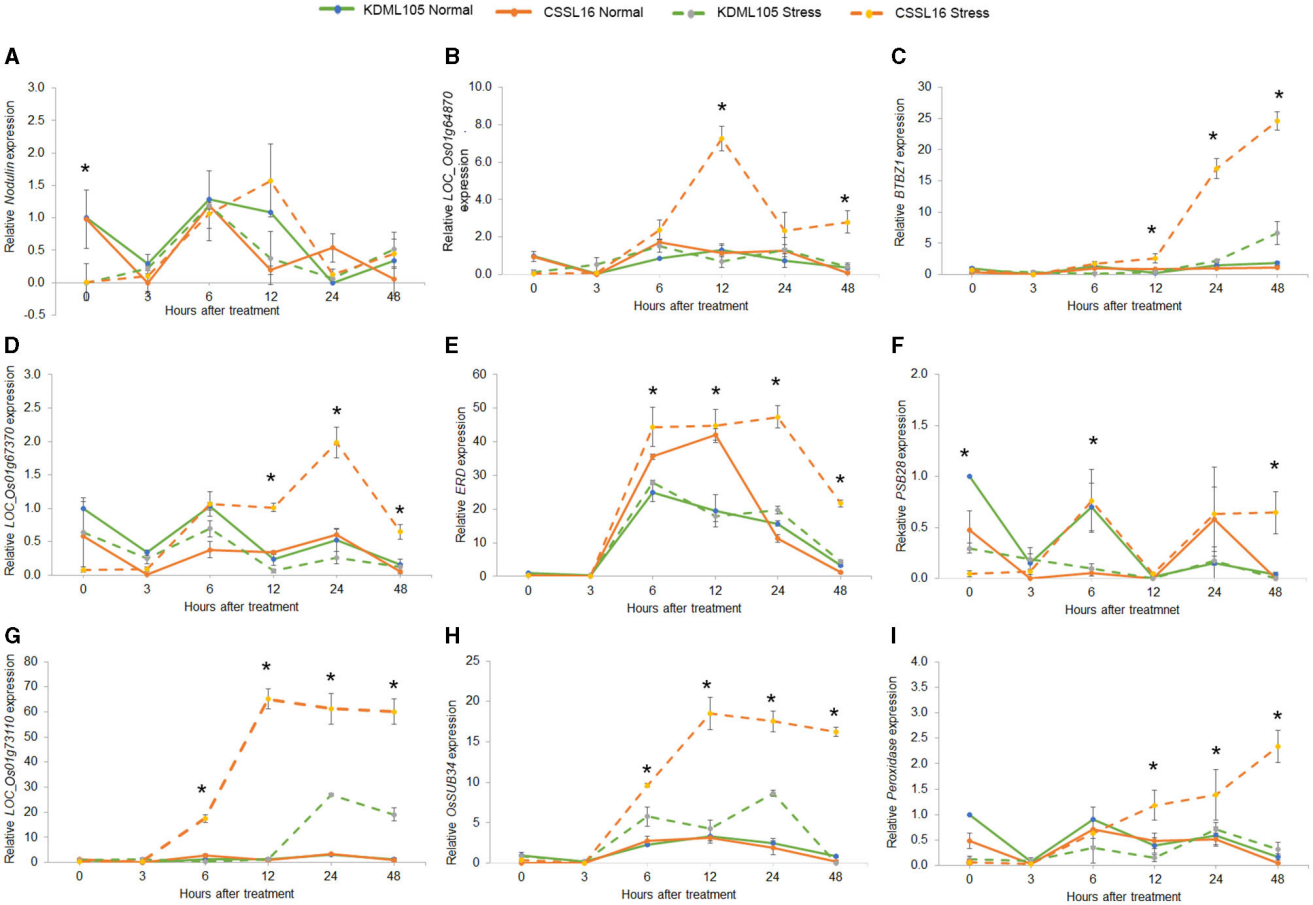
Gene expression analysis of nine candidate genes, *Nodulin* **(A)**, *Os01g64870* **(B)**, *BTBZ1* **(C)**, *Os01g67370* **(D)**, *ERD* **(E)**, *PSBS28* **(F)**, *Os01g73110* **(G)**, *Sub34* **(H)**, and *Peroxidase* **(I)** in CSSL16 and KDML105 under normal and salt stress conditions after 0, 3, 6, 9, 12, 24, and 48 h of salt stress. *indicates the significant difference among mean of the gene expression at *p* < 0.05.

To examine the salt-tolerance candidate gene expression, we used qRT-PCR to study the expression response to salt stress of the nine genes in Table 4. After growing rice seedlings for 14 days, 75 mM NaCl was added to the nutrient solution. We compared their expression in CSSL16, the salt-tolerant genotype, and in its salt-susceptible parent, “KDML105.” The comparison was performed in two sets of experiments to investigate the early (0, 3, 6, 12, 24, and 48 h after stress) and late (0, 3, and 6 days after stress) responses. After 6 days of salt stress, morphology of the plants is displayed in Figures 8A,B. For early stress responses, *OsNodulin* expression did not vary much during this period of salt stress (Figure 9A), while *LOC_Os01g64870* expression in the salt-treated CSSL16 after 12 h of salt treatment was increased to more than 7-fold higher than treated KDML105 (Figure 9B). The expression levels of *OsBTBZ1* (Figure 9C), *LOC_01g67370* (Figure 9D), and *OsPeroxidase* (Figure 9I) in the salt-treated CSSL16 were also significantly higher than those of the salt-treated KDML105 after 12 h of the treatment, while the expression levels of *OsERD* (Figure 9E), *LOC_01g73110* (Figure 9G), and *OsSub34* (Figure 9H) in CSSL16 was dramatically higher than KDML105 after 6 h of salt stress. It is worth mentioning that the expression of *OsBTBZ1, OsERD, OsSub34*, and *LOC_01g73110* was induced more than 15-fold by salt stress in the early response. The expression level of *OsPSB28* (Figure 9F) was higher in CSSL16 after 6 and 48 h of stress, but the level of expression was fluctuating and did not show much difference during this early response.

**Figure 10:**
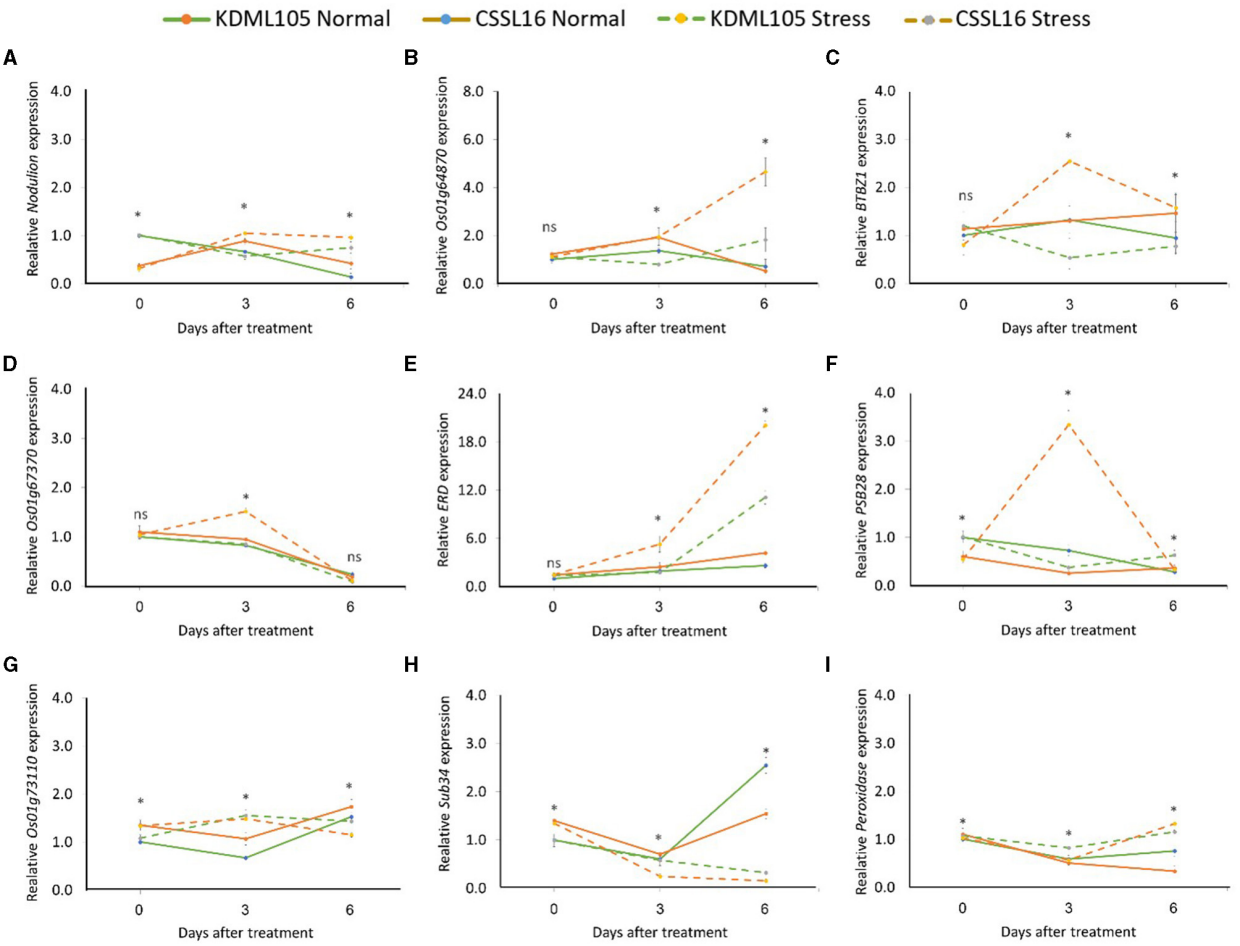
Gene expression analysis of nine candidate genes, *Nodulin* **(A)**, *Os01g64870* **(B)**, *BTBZ1* **(C)**, *Os01g67370* **(D)**, *ERD* **(E)**, *PSBS28* **(F)**, *Os01g73110* **(G)**, *Sub34* **(H)**, and *Peroxidase* **(I)** in CSSL16 and KDML105 under normal and salt stress conditions at day 0, 3, and 6 of salt stress. *indicates the significant difference among mean of the gene expression at *p* < 0.05.

For the late response, the expression of *Nodulin* (Figure 10A), *LOC_Os01g64870* (Figure 10B), *BTBZ1* (Figure 10C), *LOC_Os0167370* (Figure 10D), and *PSB28* (Figure 10F), increased significantly in CSSL16, but decreased in KDML105 at 3 days of exposure to salt stress. However, the expression of *ERD* (Figure 10E) and *LOC_Os01g73110* (Figure 10G) increased in both CSSL16 and KDML105 at 3 days of salt stress. After 6 days of salt stress, the expression of *Nodulin, LOC_Os01g64870*, and *BTBZ1* was still higher in CSSL16 compared with that of KDML105, but the expression of *LOC_Os01g73110* decreased, while the expression of *ERD* increased. After 6 days of salt stress, the expression of *ERD* increased by more than 4.5- and 4-fold in CSSL16 and KDML105, respectively. The expression of *OsSub34* was reduced by salt stress in both lines; however, this decrease was more pronounced in CSSL16 than that in “KDML105” (Figure 10H). Peroxidase increased after 6 days of salt stress in both lines (Figure 10I). The results suggest that the nine candidate genes may be involved in salt tolerance in rice.

### The Predicted Genes Have the Potentials to Function in Salt Tolerance

In order to investigate the potential of these predicted genes for functioning in salt tolerance, Arabidopsis mutant lines containing T-DNA insertion in the genes orthologous to the predicted rice genes, were analyzed for their salt responsive phenotypes. Due to the dramatically higher induction at early response of *OsBTBZ1, OsSub34*, and *LOC_01g73110*, the Arabidopsis mutants of their ortholgous genes (Table 4), namely *bt3, sbt3.3, sbt3.4*, and *at5g45310* mutants, were analyzed. Although *OsERD4* displayed high level during early induction, the *erd4* mutant was not included in this analysis because no homozygous insertion lines could be obtained. Finally, the *psb28* and *per3* mutants were included in this experiment and Col-0 wild type (WT) was used as a control.

Under normal growth condition, *sbt3.4, psb28*, and *per3* mutants showed significantly higher dry weights than WT, while the *bt3* mutant had significantly lower dry weight. The photosynthetic pigment contents were also different among these lines. The *at5g45310* mutant displayed a similar phenotype to the WT, and so did *sbt3.3*, except that *sbt3.3* had higher chl *a* content than the WT (Table 5).

**Table 5:** Dry weight per plant, chlorophyll *a*, chlorophyll *b* and carotenoid contents of 14 day-old Col-0 wild type, *bt3, sbt3.3, sbt3.4, at5g45310, psb28*, and *per3* mutants grown in MS medium or MS medium supplemented with 100 mM NaCl for 7 days.

	**Line**	Dry weight^*^ (mg/pl)	Chlorophyll a^*^ (μg·mg^**−1**^ FW)	Chlorophyll b^*^ (μg·mg^**−1**^ FW)	Carotenoid^*^ (μg·mg^**−1**^ FW)
Normal	Col-0	0.471 ± 0.062^de^	0.482 ± 0.039^b^	0.179 ± 0.028^ab^	0.149 ± 0.001^a^
	*bt3*	0.379 ± 0.023^f^	0.378 ± 0.022^d^	0.203 ± 0.003^a^	0.138 ± 0.006^bc^
	*sbt3.3*	0.496 ± 0.020^d^	0.525 ± 0.028^a^	0.189 ± 0.036^ab^	0.177 ± 0.003^a^
	*sbt3.4*	0.667 ± 0.089^b^	0.418 ± 0.011^c^	0.166 ± 0.004^b^	0.133 ± 0.007^bc^
	*at5g45310*	0.467 ± 0.039^de^	0.480 ± 0.006^b^	0.164 ± 0.005^b^	0.161 ± 0.001^ab^
	*psb28*	0.604 ± 0.072^c^	0.512 ± 0.016^ab^	0.180 ± 0.002^ab^	0.117 ± 0.005^cd^
	*per3*	0.704 ± 0.098^ab^	0.354 ± 0.033^d^	0.105 ± 0.005^c^	0.102 ± 0.016^d^
Salt stress	Col-0	0.416 ± 0.023^ef^	0.222 ± 0.009^ef^	0.068 ± 0.007^d^	0.070 ± 0.008^e^
	*bt3*	0.147 ± 0.016^g^	0.077 ± 0.013^h^	0.031 ± 0.02^c^	0.044 ± 0.021^ef^
	*sbt3.3*	0.518 ± 0.015^d^	0.213 ± 0.036^ef^	0.116 ± 0.05^c^	0.030 ± 0.046^gh^
	*sbt3.4*	0.758 ± 0.02^a^	0.231 ± 0.010^ef^	0.107 ± 0.008^c^	0.038 ± 0.005^gh^
	*at5g45310*	0.469 ± 0.015^de^	0.199 ± 0.033^f^	0.088 ± 0.004^cd^	0.058 ± 0.015^ef^
	*psb28*	0.667 ± 0.095^b^	0.243 ± 0.012^e^	0.099 ± 0.011^cd^	0.045 ± 0.008^ef^
	*per3*	0.713 ± 0.04^ab^	0.162 ± 0.017^g^	0.120 ± 0.007^c^	0.015 ± 0.004^h^

* *The values are shown in mean ± SD with three replicates. Each replicate contains 20 plants. The different letters above value represent the significantly different among means compared in the same column at p < 0.05*.

Salt stress caused dry weight reduction in the WT, but it had decreased effects on the *sbt3.3, sbt3.4, at5g45310, per3*, and *psb28* mutants. A negative effect of salt stress on dry weight was detected in the *bt3* mutant, with more than 60% reduction in dry weight. Salt stress conditions caused the reduction of photosynthetic pigments content in all lines, especially the *bt3* mutant, whose photosynthetic pigments content was decreased more than 65%. Interestingly, the carotenoid content in *sbt3.3* and *per3* mutants was dramatically decreased by salt stress (more than 80% reduction), but both mutants displayed better Chl *b* maintenance than the WT (Table 5). These changes in salt stress responses in these mutant lines, when compared to WT, suggest a role for these genes in salt stress adaptation in Arabidopsis and reinforce the hypothesis of functions of these gene families in other plant species, including rice.

Because the *bt3* mutant displayed the highest growth inhibition and photosynthesis pigment reduction and the *OsBTBZ1* gene was highly-induced under salt stress, we focused on its promoter. We compared putative regulatory sequences 2 kb base pairs upstream from the coding region of *OsBTBZ1* in the KDML105 and CSSL16 accessions analyzing it for putative regulatory cis-elements (Supplementary Figure 4).

Three ABA responsive elements (ABREs) are located within 250 base pairs upstream of the gene. Moreover, four MYC binding sites, which represent water-stress responsive elements, are located within this region, and two out of four overlapped with the ABREs. Beyond this region, −251 to −2,000 bp, 12 more MYC binding sites are found. The MYB transcription factor was also reported for water stress and salt stress regulation (review; Ponce et al., 2021). Five MYB binding sites are located in the putative regulatory sequence of *OsBTBZ1* gene.

Two elements that are found only in the putative regulatory region of CSSL16's *OsBTBZ1* gene, but not in KDML105's are an endosperm-specific element (AAAG) and GAGA-binding site. The insertion and base substitution in KDML105 eliminate the two elements found in CSSL16. This polymorphism may contribute to the difference in *OsBTBZ1* gene expression level in these two rice lines.

## Discussion

In the present study, the results of the gas exchange parameters and yield components indicated that CSSL16 was more resistant to salt stress than KDML105 at the booting stage, as it recorded higher *Pn* and yield components than KDML105 (Table 2). This was consistent with the reports of Chutimanukul et al. (2018a,b), who examined salt tolerance in rice at the seedling and vegetative stages. Salt-tolerant rice varieties can maintain their photosynthetic ability after a short period of salt stress (Moradi and Ismail, 2007); however, shoot biomass may decrease (Bhowmik et al., 2009; Krishnamurthy et al., 2009). In the present study, we documented higher stomatal conductance in CSSL16 than in “KDML105,” which may have contributed to the higher net photosynthetic rate observed in CSSL16 (Figures 1B,D). Robinson (1988) reported that stomatal conductance and transpiration rate adaptation were the most important mechanisms for salt tolerance. Although the *Pn* of the second leaves of CSSL16 was lower than the *Pn* of the second leaves of KDML105, the tiller number per plant and filled grain number of CSSL16 were higher than those of KDML105 after salt stress. These results suggest that photosynthetic activity in the flag leaves contributed more to grain filling than that of the second leaves. However, salt stress during the booting stage did affect the overall yield of the rice lines (Table 2).

Studies in various plant species have shown that salt stress results in a decrease in F_v_/F_m_ (Huang et al., 2014; Martins et al., 2020; Sun et al., 2021). A reduction of F_v_/F_m_ can be used as an indicator of photo-inhibition in stressed plants (Hichem et al., 2009). In the present study, the F_v_/F_m_ values of the flag leaves were unaffected by salt stress at the booting stage. Lisa et al. (2011) reported an increase in the expression of photosynthesis-related genes in salt tolerant rice cultivars. In the present study, photosynthesis was sustained in the CSSL16 at the vegetative stage under salt stress and this may be due to the higher expression of the *PsbS1* gene encoding the chlorophyll binding protein in photosystem II (Chutimanukul et al., 2018b). Contrarily, the “KDML105” rice had the lowest *Pn*, suggesting that it was the most susceptible compared with the other lines. Pi refers to the quantum efficiency of primary photochemistry, the concentration of reaction centers, and excitation energy conversion in electron transport (Melis, 1999; Strasser et al., 2000). At the booting stage, CSSL14 and CSSL16 had higher Pi values under salt stress (Figure 3F), indicating that they were able to maintain the quantum efficiency of primary photochemistry.

A comparison of the three methods of transcriptomic analysis showed that WGCN identified the highest number of salt tolerance candidate genes, while CC identified the lowest number of candidate genes. Among the 92 genes identified by the three methods, nine genes contained SNPs in CSSL16 and KDML105. The expression level of the nine genes was different in CSSL16 and KDML105, consistent with the notion that they may be involved in regulating salt tolerance. Moreover, seven of the genes were located in the salt tolerance QTL reported by Kanjoo et al. (2012) (Figure 1).

The expression analysis of these nine genes within 48 h (Figure 8) showed much higher induction in *OsBTBZ1, OsERD, LOC_Os01g73110*, and *OsSUB34* genes, when compared to the expression at later stages (Figure 9), suggesting that these four genes may function in the early response to salt stress. Therefore, we have tried to investigate the roles of these genes in salt stress tolerance by using the Arabidopsis mutant with T-DNA insertion in these orthologous genes. Unfortunately, we cannot obtain homozygous of Arabidopsis mutant with T-DNA insertion in *ERD4* at this moment. We also investigate the Arabidopsis mutant with T-DNA insertion in *PSB28* and *Per3* gene. The decrease in photosynthetic pigments and changes in dry weight response in the mutant lines support the role of the genes in salt tolerance.

Some of the nine genes were reported to be involved in stress responses. *LOC_Os01g61010 (Nodulin)* encodes a member of a family of highly conserved proteins involved in regulating membrane transporters. Wallace et al. (2006) found that *Nodulin* contributed to water permeability under osmotic stress in soybean. Moreover, *Nodulin* stimulated phosphorylation to regulate the process of cellular transport during osmotic adaptation in soybean exposed to salt or drought stress (Guenther et al., 2003). *LOC_Os01g73110* has not been characterized. However, Sircar and Parekh (2015), who investigated the function of *LOC_Os01g73110* using the AraNet and RGAP database identified its homolog in Arabidopsis as *AT5G45310*, whose product is involved in the biosynthesis of abscisic acid (ABA). *LOC_Os01g67370* Arabidopsis ortholog, *AT3G59300*, encodes a pentatricopeptide-repeat (PPR) superfamily protein. Some PPR proteins in Arabidopsis have been associated with abiotic stress responses, including oxidative stress and ABA responses (Liu et al., 2016). *PSB28* was found to be associated with photosystem II reaction center and water splitting in light-dependent reactions. Suorsa and Aro (2007) reported the molecular function of *PSB28*. The *PSB28* rice mutant identified from the T-DNA insertion population exhibited a pale green plant (Jung et al., 2008). The expression of *PSB28* was reduced under water stress and heat stress in tomato seedlings (Zhang et al., 2018) and *Populus tomentosa* (Ren et al., 2019), respectively. Moreover, Kosmala et al. (2009) found that expression of the *PSB28* gene responded to cold stress in *Festuca pratensis*. These results indicated that PSII and PSI were suppressed under stress conditions. Consequently, the accumulation of *PSB28* might enhanced the electron transport rate and photochemical efficiency.

*OsSub34* encodes a subtilisin protein associated with serine peptidase. Subtilisin contributes to plant responses under biotic and abiotic stress, organ abscission, senescence, and programmed cell death (Schaller et al., 2018). In rice, *LOC_Os06g46799* encodes a peroxidase precursor that is highly responsive to various abiotic stress stimuli and plays an important role in the regulation of reactive oxygen species (ROS) by converting H_2_O_2_ to water (Hiraga et al., 2001). Hiraga et al. (2001) identified a group of genes that encodes redox regulation-related proteins, including ascorbate peroxidase, peroxidase precursor, glutathione synthetase, and glutathione S-transferase, in rice exposed to drought stress. Moreover, Chutimanukul et al. (2019) reported that CSSL16 had higher peroxidase activity than that did KDML105 under salt stress at the seedling stage, which supports the role of *LOC_Os06g46799* in the present study.

*BTBZ1* and *ERD* are proposed to be the genes with the highest correlation with salt tolerance in the rice lines, as both were predicted by three methods of gene co-expression network analysis. Additionally, *BTBZ1* and *ERD* contained SNPs in CSSL16 and KDML105 and both genes were located in the salt/drought QTL previously identified by Kanjoo et al. (2011). Consistent with a joint requirement for both genes for optimal stress tolerance, CSSL10 and CSSL14 carry, respectively, either the *BTBZ1* or the *ERD* allele of DH212 and neither displays the full tolerance phenotype of CSSL14. *BTBZ1* belongs to the Bric-A-Brac/Tramtrack/Broad Complex (BTB) protein superfamily (subfamily C1) and contains a TAZ zinc finger and calmodulin-binding domain. The homologous gene in Arabidopsis, *AtBT1*, encodes a nuclear CaM-binding protein. The expression of *AtBTs* can be triggered by stress stimuli (Du and Poovaiah, 2004). BTB-ZF proteins are known as the POK, POZ, and Krüppel zinc finger proteins (Deweindt et al., 1995). Moreover, Stogios et al. (2005) reported that the *BTB* domain is a protein-protein interaction motif that is involved in cellular functions, including transcriptional regulation, cytoskeleton dynamics, ion channels, and targeting proteins for ubiquitination. Moreover, *BTB-ZF* genes constitute a supergene family encoding proteins that are thought to be transcription factors. Additionally, the analysis of protein-protein interactions from the Predicted Rice Interactome network (PRIN) indicated that the *BTBZ1* protein interacted with a cullin protein (*LOC_Os02g51180*), which may be involved in the degradation of the target protein through the ubiquitin/proteasome pathway (Figueroa et al., 2005). Several reports have described the important role of BTB proteins in developmental programs, defense, and abiotic stress responses (Weber and Hellmann, 2009; Prasad et al., 2010). Nutrient, stress, and hormone responses were regulated by *AtBT2* in Arabidopsis (Mandadi et al., 2009). However, an ortholog of the *BTBZ* gene in Arabidopsis (AT1G05690) was involved in plant development (Robert et al., 2009).

*ERD* was associated with early response to dehydration, which could be rapidly induced during drought stress and other abiotic stresses. *ERD* is a member of a large gene family, whose protein products are associated with triphosphate (ATP)-dependent proteases, heat shock proteins (HSPs), membrane proteins, proline, sugar senescence-related genes, chloroplasts, biosynthesis, protein transporters, dehydrogenase, and ubiquitin extension proteins (Kiyosue et al., 1994; Taji et al., 1999; Simpson et al., 2003). Borah et al. (2017) reported that “Dhagaddeshi rice,” a drought-tolerant cultivar, had higher expression levels of *ERD1* and responded faster than the susceptible cultivar (IR20) to drought stress. Moreover, Liu et al. (2009) found that the *ERD4* gene played a key role in the adaptation of maize to the early stages of stress and enhanced the plant's tolerance to abiotic stress conditions. In transgenic tobacco, the overexpression of *ERD15* increased the efficiency of PSII (F_v_/F_m_) through the protection of cellular membranes (Ziaf et al., 2011). Additionally, transgenic Arabidopsis plants overexpressing the *BjERD4* gene from *Brassica juncea* displayed increased tolerance to salt stress and drought, while the *Bjedr4* knockdown lines were susceptible to salt and drought stress (Rai et al., 2016). Therefore, *ERD* may contribute to salt tolerance in rice.

The *bt3* Arabidopsis mutant showed the highest reduction in growth and photosynthesis pigment content, while in rice, more than 20-fold induction of *OsBTBZ1* gene was detected after 48 h of salt stress treatment. This is consistent with the *cis*-regulatory elements found in the putative *OsBTBZ1* promoter (Supplementary Figure 4), which include 3 ABREs, 5 MYB binding sites, and 16 MYC binding sites. Many MYB proteins regulate salt tolerance through regulation of the ABA signaling pathway (review: Wang et al., 2021). Both MYB and MYC proteins function as the transcriptional activators in ABA signaling in Arabidopsis (Abe et al., 2003). Together with this literature information our finding support an *OsBTBZ1* contribution to salt tolerance phenotype of CSSL16. The upstream region of *OsBTBZ1* consists of multiple *ERD* binding sites. We identified *OsERD* as one of the key genes because it was highly induced prior to *OsBTBZ1* (45-fold induction) in CSSL16, while it was up-regulated only 25-fold in KDML105. Therefore, the interaction between *OsBTBZ1* and *OsERD* and their involvement in salt tolerance in rice should be further characterized.

Due to insertion and base substitution in the putative promoter region of *OsBTBZ1* in KDML105, GAGA binding site was detected only in CSSL16. In Arabidopsis, bHLH34 binds to GAGA element and is involved in ABA and salinity response (Min et al., 2017). Moreover, rice Trithorax factor ULTRAPETALA 1 (OsULT1) was found to bind the promoter region of the *OsDREB1b* gene during transcriptional activation. The binding of OsULT1 to GAGAG elements decreases tri-methylation of lysine 27 on histone H3 (H3K27me3), which antagonizes the transcriptional repression effect of H3K27me3, favoring transcriptional activation of the gene (Roy et al., 2019). A similar phenomenon may occur in the regulation of *OsBTBZ1* leading to the higher expression in CSSL16 than KDML105.

**Figure 11:**
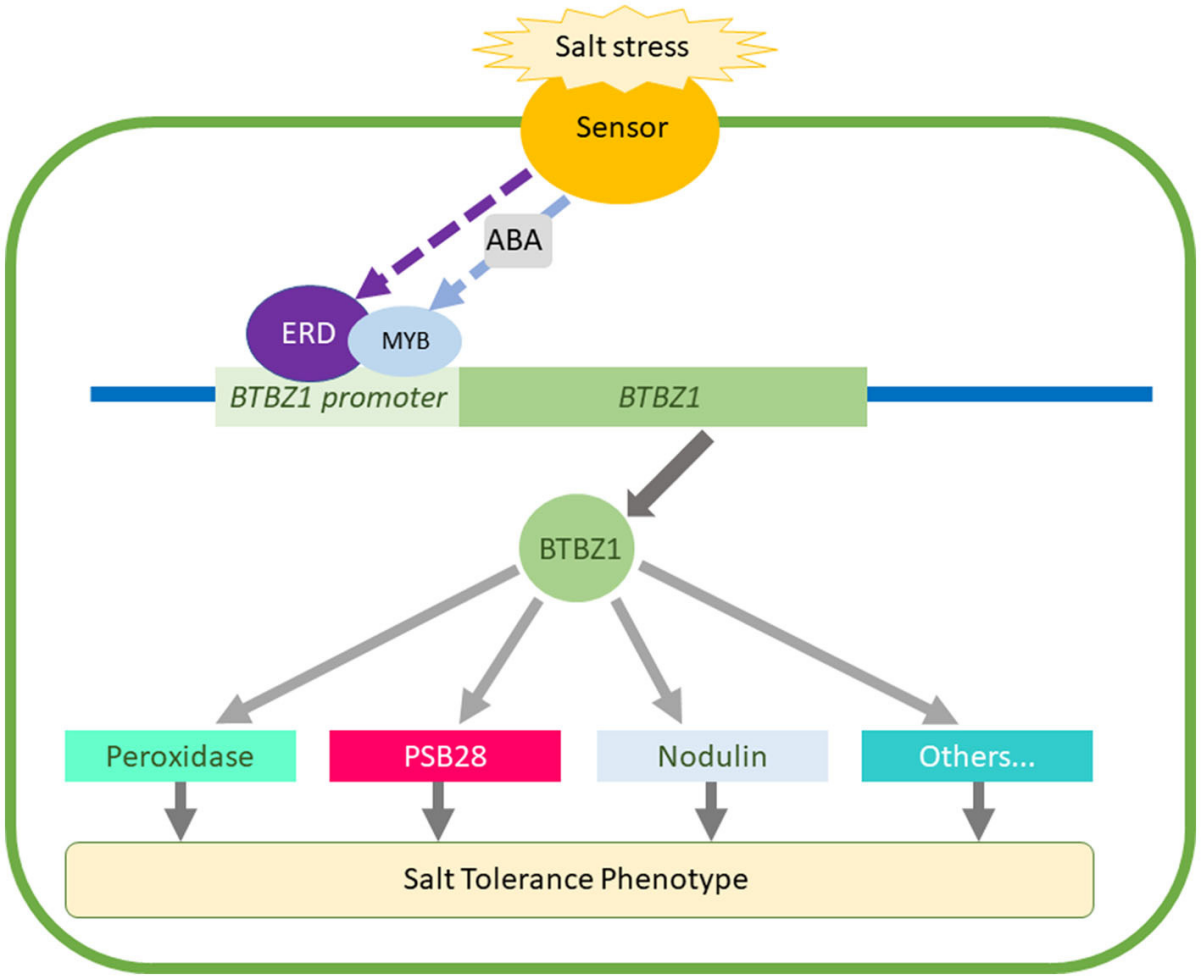
Hypothetical model for the function of the predicted genes obtained from the combining of genome and gene co-expression network analysis.

Considering all our findings, we hypothesize that the predicted key regulatory genes in the network reported here coordinate a response that makes the rice plants more tolerant to salt stress. The earlier and higher expression of *LOC_Os01g64870, OsBTBZ1, LOC_Os01g67370, OsERD, LOC_Os01g73110, OsSUB34*, and *OsPeroxidase* in CSSL16 leads to higher salt tolerance when compared to KDML105. Further investigations should be performed to validate this hypothesis in the future. Based on the ERD binding site in *OsBTBZ1* putative promoter, we hypothesize that the ERD protein regulates *OsBTBZ1* gene expression and regulates other genes such as PSB28, and Peroxidase. The proposed model for this hypothesis is shown in Figure 11.

According to the comparison of the predicted alleles from chromosome 1 of DH212 in CSSLs, 3 candidate alleles from DH212, *Nodulin* (*LOC_Os01g61010*), *LOC_Os01g64870*, and *BTBZ1* (*LOC_Os01g66890*) are located in CSSL10, while CSSL14 contains another 3 candidates from DH212, which are *PSB28* (*LOC_Os01g71190*), *ERD* (*LOC_Os01g72210*), and *LOC_Os01g73110*. The salt tolerance phenotype of CSSL16 was significantly higher than CSSL10 and CSSL14 in all stages, seedling (Chutimanukul et al., 2018a, 2019), vegetative (Chutimanukul et al., 2018b) and booting stages. Therefore, we explicitly propose that the whole QTL in this region is necessary for salt tolerance in rice.

## Conclusion

In the present study, we demonstrate an effective transcriptomic approach for identifying genes regulating salt tolerance in rice using two rice lines with close genetic relationships, but different salt tolerance ability. Combining GCN, CC, and WGCN analyses with available SNP information, we identified nine genes involved in salt tolerance in rice. Under salt stress, the expression levels of the nine genes differed in the two rice lines. Moreover, most of the genes were involved in abiotic stress responses. Therefore, we can conclude that the combination of the three methodologies for transcriptome analysis, GCN, CC, and WGCN with SNP information is an effective approach for the identification of genes involved in abiotic stress tolerance and it can support the identification of appropriate QTL for salt tolerance improvement.

## Data Availability Statement

The datasets presented in this study can be found in online repositories. The names of the repository/repositories and accession number(s) can be found in the article/Supplementary Material.

## Author Contributions

SC, LC, TT, and KP: study conception and design. PC, PM, MS, and TBS: data collection. PC, TBS, PM, KP, SC, and TB: analysis and interpretation of results. PC, TB, KP, LC, and SC: draft manuscript preparation. All authors reviewed the results and approved the final version of the manuscript.

## Conflict of Interest

The authors declare that the research was conducted in the absence of any commercial or financial relationships that could be construed as a potential conflict of interest.

## Publisher's Note

All claims expressed in this article are solely those of the authors and do not necessarily represent those of their affiliated organizations, or those of the publisher, the editors and the reviewers. Any product that may be evaluated in this article, or claim that may be made by its manufacturer, is not guaranteed or endorsed by the publisher.

## Supplementary Material

Supplementary File 1 Differentially Expressed Genes in seedling leaves, flag leaves, second leaves.

Supplementary Figure 1 The gene set enrichment analysis of differentially expressed genes in seedlings when compared between the transcriptome of seedlings grown under normal (0 day) condition and after 75 mM NaCl treatment for 2 days in term of biological process by using ClueGO tool.

Supplementary Figure 2 The gene set enrichment analysis for biological process of differentially expressed genes when compared between the transcriptome of second leaves from rice plants grown under normal (0 day) condition and after 75 mM NaCl treatment for 3 days at booting stage with ClueGO tool.

Supplementary Figure 3 The gene set enrichment analysis for biological process of differentially expressed genes when compared between the transcriptome of flag leaves from rice plants grown under normal (0 day) condition and after 75 mM NaCl treatment for 3 days at booting stage with ClueGO tool.

Supplementary Figure 4 The putative regulatory element analysis of *OsBTBZ1* gene from CSSL16 and KDML105.

Supplementary File 2 The number of differentially expressed genes in leaves at seedling stage, second leaf, and flag leaf at booting stage under salt stress condition.

Supplementary File 3 List of significant candidate genes identified by gene co-expression network, clustering co-efficient and weighted gene co-expression network.

## Abbreviations

CC: Clustering coefficients
*Ci*: internal CO_2_ concentration
CSSL: chromosome segment substitution line
*E*: transpiration rate
F_v_/F_m_: maximum PSII efficiency
GCN: Gene co-expression network
GO: Gene ontology
*g*_*s*_: stomatal conductance
KDML105: Khao Dawk Mali 105
PAR: Photosynthetically active radiation
Pi: Photosynthetic performance index
*Pn*: Net photosynthetic rate
PSI: Photosystem I
PSII: Photosystem II
RNA-seq: RNA sequencing
ROS: Reactive oxygen species
SES: Standard Evaluation System
WGCN: Weighted gene co-expression network.
