# Supplementary figures and images for: Combining Genome and Gene Co-expression Network Analyses for the Identification of Genes Potentially Regulating Salt Tolerance in Rice
Source: Front Plant Sci. 2021 Aug 26;12:704549. doi: 10.3389/fpls.2021.704549 (PMC8427287; doi:10.3389/fpls.2021.704549)

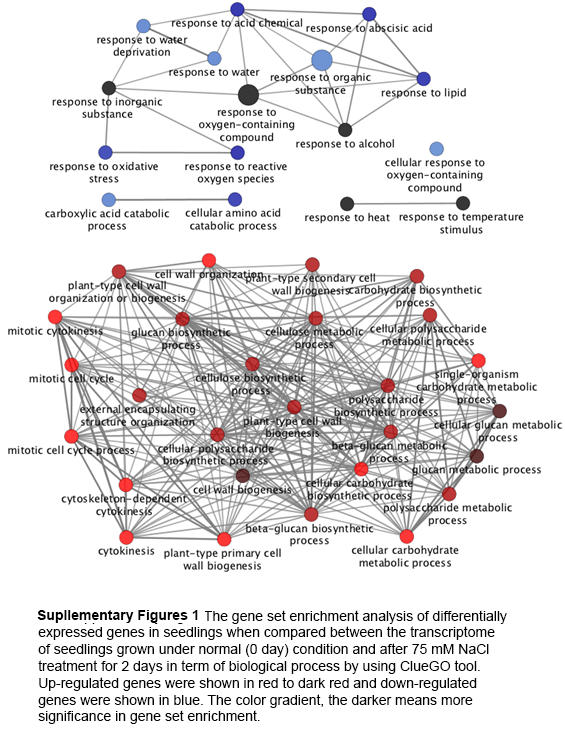

Supplement: Supplementary Figure 1 — The gene set enrichment analysis of differentially expressed genes in seedlings when compared between the transcriptome of seedlings grown under normal (0 day) condition and after 75 mM NaCl treatment for 2 days in term of biological process by using ClueGO tool. [file Image_1.tif]

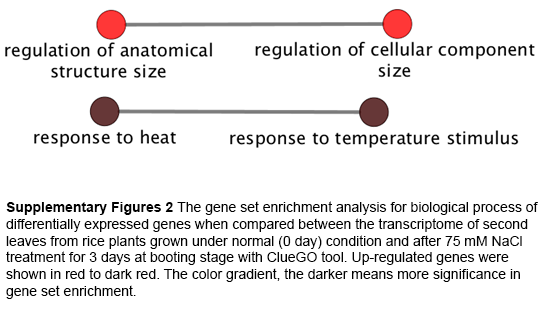

Supplement: Supplementary Figure 2 — The gene set enrichment analysis for biological process of differentially expressed genes when compared between the transcriptome of second leaves from rice plants grown under normal (0 day) condition and after 75 mM NaCl treatment for 3 days at booting stage with ClueGO tool. [file Image_2.tif]

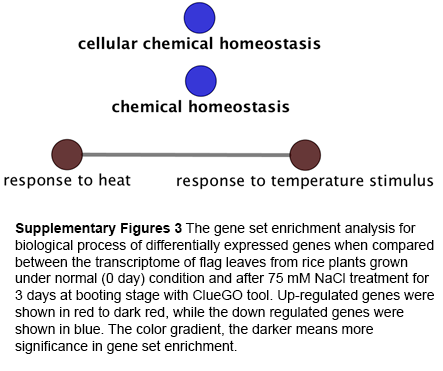

Supplement: Supplementary Figure 3 — The gene set enrichment analysis for biological process of differentially expressed genes when compared between the transcriptome of flag leaves from rice plants grown under normal (0 day) condition and after 75 mM NaCl treatment for 3 days at booting stage with ClueGO tool. [file Image_3.tif]

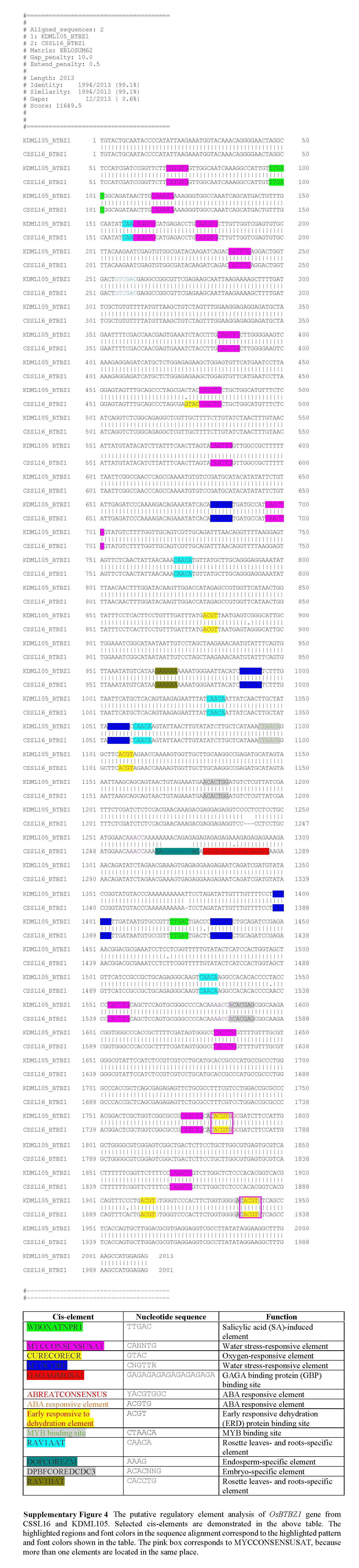

Supplement: Supplementary Figure 4 — The putative regulatory element analysis of OsBTBZ1 gene from CSSL16 and KDML105. [file Image_4.tif]
